# A Glycine Insertion in the Estrogen-Related Receptor (*ERR*) Is Associated with Enhanced Expression of Three Cytochrome P450 Genes in Transgenic *Drosophila melanogaster*

**DOI:** 10.1371/journal.pone.0118779

**Published:** 2015-03-11

**Authors:** Weilin Sun, M. Carmen Valero, Keon Mook Seong, Laura D. Steele, I-Ting Huang, Chien-Hui Lee, John M. Clark, Xinghui Qiu, Barry R. Pittendrigh

**Affiliations:** 1 Department of Entomology, University of Illinois at Urbana-Champaign, Urbana, Illionois, 61801, United States of America; 2 Chung Hwa University of Medical Technology, Tainan, Taiwan, R. O. C.; 3 Department of Veterinary & Animal Science, University of Massachusetts, Amherst, Massachusetts, 01003, United States of America; 4 State Key Laboratory of Integrated Management of Pest Insects and Rodents, Institute of Zoology, Chinese Academy of Sciences, Beijing, China; University of Geneva, SWITZERLAND

## Abstract

Insecticide-resistant *Drosophila melanogaster* strains represent a resource for the discovery of the underlying molecular mechanisms of cytochrome P450 constitutive over-expression, even if some of these P450s are not directly involved in the resistance phenotype. For example, in select 4,4’-dichlorodiphenyltrichloroethane (DDT) resistant strains the glucocorticoid receptor-like (GR-like) potential transcription factor binding motifs (TFBMs) have previously been shown to be associated with constitutively differentially-expressed cytochrome P450s, *Cyp12d1*, *Cyp6g2* and *Cyp9c1*. However, insects are not known to have glucocorticoids. The only ortholog to the mammalian glucocorticoid receptor (GR) in *D. melanogaster* is an estrogen-related receptor (*ERR*) gene, which has two predicted alternative splice isoforms (*ERRa* and *ERRb*). Sequencing of *ERRa* and *ERRb* in select DDT susceptible and resistant *D. melanogaster* strains has revealed a glycine (G) codon insertion which was only observed in the ligand binding domain of ERR from the resistant strains tested (*ERR-G*). Transgenic flies, expressing the *ERRa-G* allele, constitutively over-expressed *Cyp12d1*, *Cyp6g2* and *Cyp9c1*. Only *Cyp12d1* and *Cyp6g2* were over-expressed in the *ERRb-G* transgenic flies. Phylogenetic studies show that the G-insertion appeared to be located in a less conserved domain in ERR and this insertion is found in multiple species across the Sophophora subgenera.

## Introduction

Resistance to insecticide is a major challenge for pest control and also an important man-made micro-evolutionary force [1,2]. Resistance is also a system for studying microevolution and environmental adaptation [2,3]. DDT (4,4’-dichlorodiphenyltrichloroethane) resistance in *Drosophila melanogaster* has been used for the study of the evolution of insecticide resistance. Metabolic insecticide resistance is an important form of resistance and is often associated with constitutive over-expression of (or, in some cases, structural changes in) cytochrome P450s as well as glutathione-S-transferases (GSTs), esterases, or a combination of these genes [4–6].

Much of the emphasis in the pesticide resistance community has been on the discovery of which genes confer the resistant phenotype, however, pesticide resistant strains also represent a resource to investigate the molecular mechanisms by which transcription of P450s occurs, regardless if these genes play any role in resistance. For example, moderate- to high-levels of metabolic DDT resistance in *D. melanogaster* is generally considered to be polygenic [5–10]. However, metabolic DDT resistance is not a single phenotype; the lethal concentration 50 (LC_50_) for DDT varies considerably across pesticide-resistant *D. melanogaster* strains [10]. The two metabolically insecticide-resistant fly strains, *Wisconsin* and *91-R*, show very different LC_50_s when bioassayed with DDT; *91-R* is far more resistant to DDT than *Wisconsin*. Several reports in the literature have shown that constitutive over-expression of *Cyp12d1*, *Cyp6a2* and *Cyp6g1* occurs in some DDT-resistant strains [6,11,12]. However, a recent toxicokinetic analysis of *91-R* revealed that oxidative P450s likely cause little direct metabolic resistance, with reduced penetration, increased reductive dechlorination and enhanced excretion playing more dominant roles [13]. It is unlikely, therefore, that these P450s play a critical role in moderate- to high-levels of DDT resistance through DDT metabolism, however, they are constitutively over-transcribed and little is known about the regulation of the expression. Plapp [14] hypothesized that mutated trans-regulatory factors might be involved in the constitutive over-expression of detoxification genes. Recently, it has been shown that there is constitutive activation of the Nrf2/Keap1 pathway in DTT-resistant strains and that this might cause about 20% of the genes (including several P450s) to be differentially expressed in *91-R* strain [15]. To date, only the constitutively over-expression of *Cyp6a2* has been associated with an allele containing an intact Nrf2/Maf-binding-site [16]. The mechanism(s) activating this pathway is not fully understood and it is difficult to address because there are multiple steps that can be regulated.

Recently, Qiu and collaborators [12] used a potential transcription factor binding motif (TFBM) analysis of the genes differentially transcribed in the two DDT-resistant strains, *Wisconsin* and *91-R* found that for all the genes coding for detoxification enzymes a glucocorticoid receptor-like (GR-like) motif was present. The glucocorticoid receptor (GR) is a versatile nuclear receptor (NR) of the family NR3 that in mammalians regulates genes controlling metabolism, development and immunity. Specifically, GR has been implicated in xenobiotic-induced expression of several cytochrome P450 enzymes [17]. Insects are not known to have GRs, however, the GR’s closest NR3 member in *D. melanogaster* is the estrogen-related receptor (*ERR*, CG7404, FBgn0035849), of which there are two known transcripts, *ERRa* and *ERRb*.

In the current study, we demonstrated that *ERRa* and *ERRb* isoforms, containing a G codon insertion caused constitutively over-expression of *Cyp12d1*, *Cyp6g2* and *Cyp9c1* as transgenes. The region in the *ERR* gene where the G insertion occurs appeared to be variable across *D. melanogaster* strains and this insertion was observed in other *Drosophila* species in the Sophophora subgenera.

## Materials and Methods

### *Drosophila melanogaster* strains

Three *D. melanogaster* strains were used for the initial sequencing of *ERRa* and *ERRb* cDNAs: *Canton-S* (susceptible to DDT), *Wisconsin* (moderately resistant to DDT), and *91-R* (highly resistant to DDT). Detailed descriptions of these fly strains, as well as the *91-C* (susceptible to DDT) strain, rearing conditions, and sample preparation were given in [18] and [6]. The LC_50_s for *Canton-S*, *Wisconsin* and *91-R* are given in Festucci-Buselli et al [10]. Populations of *91-C* and *91-R* strains have been maintained in the Pittendrigh laboratory for over a dozen years. For the transgenic lines *w* ^*1118*^ was used. In addition to the above strains, another 53 wild-type lines of *D. melanogaster* were obtained from the Bloomington *Drosophila* Stock Center and were genotyped for *ERR*. A detailed description of these fly strains is given in [19].

### *ERR* transcript sequencing and cloning

Total RNA from three strains (*Canton-S*, *Wisconsin and 91-R*) were used to synthesize full-length cDNA with oligo dT by using a cDNA Cycle kit (Invitrogen, CA). Gene-specific primers were used to amplify the full-length cDNA for all three strains (S1 Table). The amplified cDNA-specific products were purified using a PCR clean-up kit (Qiagen, CA) and directly sequenced with three forward and three reversing primers to cover the full length. All sequences were assembled and compared by using Vector NTI (Invitrogen, CA).

### Absolute real time quantitative (RT-qPCR) for transcript abundance of *ERRa* and *ERRb*

In order to evaluate the differences in transcript abundances between *ERRa* and *ERRb* in the *Canton-S*, *Wisconsin*, and *91-R* lines, absolute quantitative real-time PCR (RT-qPCR) was performed. Gene-specific primers were designed at the UTR of *ERRa* and *ERRb* (S1 Table). The single-stranded cDNA was synthesized using the GoScript reverse transcription system from Promega. Real-time PCR was performed at an annealing temperature of 58°C, which had been optimized by gradient RT-qPCR. For each gene, one single amplification product was confirmed by a single peak melting curve and a single band on an agarose gel. The PCR amplicon for each gene was purified using a Qiagen PCR clean-up kit and sequenced to insure correct amplification. Amplicon sizes were 125 bp and 131 bp for *ERRa* and *ERRb*, respectively. The purified amplicons were quantified using a Nanodrop 1000 spectrophotometer and tenfold dilution series (from 100 pM to 0.1 fM) were prepared for each as standards for RT-PCR analysis. Six biological replicates of adult flies (3 days old) of mixed sexes for *Canton-S*, *Wisconsin*, and *91-R* strains were tested. The absolute expression level difference between *ERRa* and *ERRb* was compared within each fly strain by using paired *t*-test since *ERRa* and *ERRb* transcripts were from the same cDNA reaction.

### Transgenic flies

Full length *ERRa* and *ERRb* cDNA from the *91-R* strain was cloned into a *pCR2*.*1* Topo vector (Invitrogen, CA). Individual clones were sequenced to find an open reading frame and correct amino acid sequences for *ERRa* and *ERRb*. The selected clones for both *ERR* transcripts were sub cloned into a pUAST vector. Transgenic flies were generated by the Best Gene Inc (Chino Hills, CA) using *w* ^*1118*^ strain. The driver strain (*y* ^1^ w*; P{*Act5C*-*Gal4-w*}*E1*/*CyO*) was obtained from Bloomington *Drosophila* Stock Center.

### Relative quantitative real time PCR (RT-qPCR)

To compare the effects of cloned *ERRa* and *ERRb* genes on P450 transcription patterns from the *Cantons-S* and *91-R* strains, we used transgenic flies before crossing (F_0_) as controls. For each gene and/or treatment combination, three biological replicates (RNA samples) were prepared. Three-day-old flies were collected for RNA extraction. RNA was extracted from 30 flies (1:1 male/female ratio) using the Qiagen RNeasy kit (Qiagen, Valencia, CA, USA) with an “on-column” DNase digestion procedure. The first-strand cDNA was synthesized by using 0.5 μg of total RNA with GoScript Reverse Transcription System from Promega (Madison, WI) in a 20 μl reaction volume with random primers. A StepOne plus real-time PCR system from Applied Biosystems (Carlsbad, CA) were used to run qRT-PCR with GoTaq qPCR master mix from Promega (Madison, WI). For each cDNA, three RT-qPCR reactions were performed. All cDNA samples were equalized with *rp49* gene as reference before target genes were tested. The average threshold cycle (Ct) for each cDNA was calculated by the StepOne software. The relative expression levels were calculated as given in [20] by using *rp49* as the reference gene. Statistical analysis was done on target gene delta Ct after adjusted by *rp49* Ct in SAS software (SAS Institute Inc., Cary, NC, USA) with a GLM model and contrast statements. All the primers for the assayed genes are listed in S1 Table.

### The structure analyses of ERRa and ERRb proteins

The tertiary structures of the isoforms ERRa and ERRb with and without the G insertion were predicted by SWISS-MODEL Workplace (http://swissmodel.expasy.org/workspace/) automated mode [21]. The tertiary structures with and without the G insertion were superimposed for both ERRa and ERRb respectively by inputting Protein Data Bank (PDB) files into the server (http://mspc.bii.a-star.edu.sg/minhn/click.html; [22]).

### Analysis of *ERR* across *D. melanogaster* strains

Genomic DNA was extracted from 10 to 15 adult flies using the DNeasy Blood and Tissue Kit (Qiagen, Valencia, CA). DNA was quantified by spectrophotometry using Nanodrop 1000 (Thermo Scientific, Wilmington, DE). Based on *D. melanogaster* reference genome (NT_037436.3), primers were designed to amplify a region of the ligand binding domain (LBD) of *ERR* gene (S1 Table). The LBD region was amplified from 100 ng of genomic DNA, 1.25 U of Go Taq (Promega Corporation, Madison, WI) with 1 × Promega Mg^2+^-free buffer, 1.5 mM of MgCl_2_, 200 μM of each dNTP and a final concentration of 200 nM of primers in a 25 μl volume. Thermal cycling started at 94°C for 5 min; followed by 35 cycles of 94°C for 30 s, annealing at 59°C for 30 s and extension at 72°C for 1 min and finished with a final extension at 72°C for 7 min. The PCR products were purified with Qiaquick PCR purification kit (Qiagen, Valencia, CA) and sequenced at the Core Sequencing Facility at University of Illinois at Urbana-Champaign.

### Analysis of *ERR* across *Drosophila* species

The orthologoues *ERR* sequences of 12 *Drosophila* species were downloaded from the Hierarchical Catalog of Orthologs (OrthoDB http://cegg.unige.ch/orthodb6): *D. ananassae* (FBgn0101695), *D. erecta* (FBgn0107165), *D. grimshawi* (FBgn0123455), *D. mojavensis* (FBgn0139382), *D. persimilis* (FBgn0160156), *D. pseudoobscura* (FBgn0080322), *D. sechellia* (FBgn0179823), *D. simulans* (FBgn0184734), *D. virilis* (FBgn0200124), *D. willistoni* (FBgn0212838), and *D. yakuba* (FBgn0237681). Sequence alignments were performed with Clustal Omega (EMBL-European Bioinformatics Institute, Cambridge, UK). The phylogeny of *ERR* was inferred by Maximum Likelihood (ML) test using the [23] JTT amino acid substitution model (MEGA5.2) [24].

## Results

### Sequencing and expression of *ERR* transcripts from *Canton-S*, *Wisconsin*, and *91-R*

The sequences for *ERRa* and *ERRb* transcripts were identical except for an extra 12 amino acids at 5’ end of *ERRb* (S1 Fig.). The *ERRa* and *ERRb* sequences from *Canton-S* strain were identical to the sequences in the GenBank. Sequencing of *ERRa* and *ERRb* from *91-R* revealed an insertion of three nucleotides that resulted in a G amino acid insertion at position 282 of the protein that was not found in *Canton-S* or the sequences in the GenBank. The sequencing for *Wisconsin* revealed heterozygosity for the G codon insertion in both *ERRa* and *ERRb*. As shown in S1 Fig., the insertion was in the same relative location for both *ERRa* and *ERRb*. Absolute RT-qPCR of *ERRa* and *ERRb* from the *Canton-S*, *Wisconsin* and *91-R* strains revealed that, in all three aforementioned strains, *ERRa* transcripts were 3.4 to 5.4 times more abundant than that found for *ERRb* (Table 1).

**Table 1:** Expression levels of *ERRa* and *ERRb* in three *Drosophila* strains by absolute RT-qPCR.

	*91-R* (±SE)	*Wisconsin* (±SE)	*Canton-S* (±SE)
*ERRa*, fM	2.72 (0.16)	1.50 (0.23)	1.21 (0.10)
*ERRb*, fM	0.50 (0.16)	0.44 (0.23)	0.24 (0.10)
**p-value**	**0.00004**	**0.006**	**0.0002**

The units are femtomole (fM) per L in total RNA.

### *ERR* expression in three different strains

**Fig 1:**
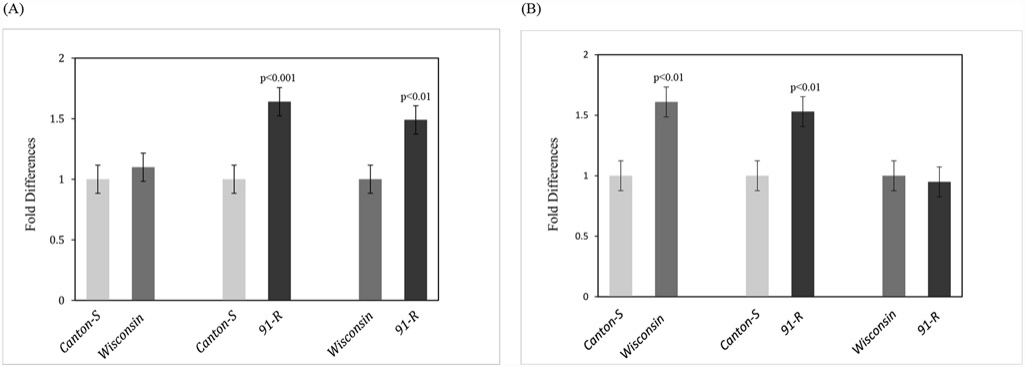
Expression levels of *ERRa* and *ERRb*. Expression of (A) *ERRa* and (B) *ERRb* transcripts in *Canton-S*, *Wisconsin* and *91-R* by relative qRT-PCR.

As shown in Fig. 1A, *ERRa* transcript level was not different between *Canton-S* and *Wisconsin*. The *ERRa* transcript level was significantly higher in *91-R* compared to the transcripts either in *Canton-S* or *Wisconsin*. For *ERRb*, the transcript abundance levels were significantly higher in both *Wisconsin* and *91-R* as compared to *Canton-S* and no difference was observed between them (Fig. 1B). However, the highest relative difference for either *ERRa* or *ERRb* was around 50%.

### *ERR* transgenic flies over-transcribe selected P450s

**Fig 2:**
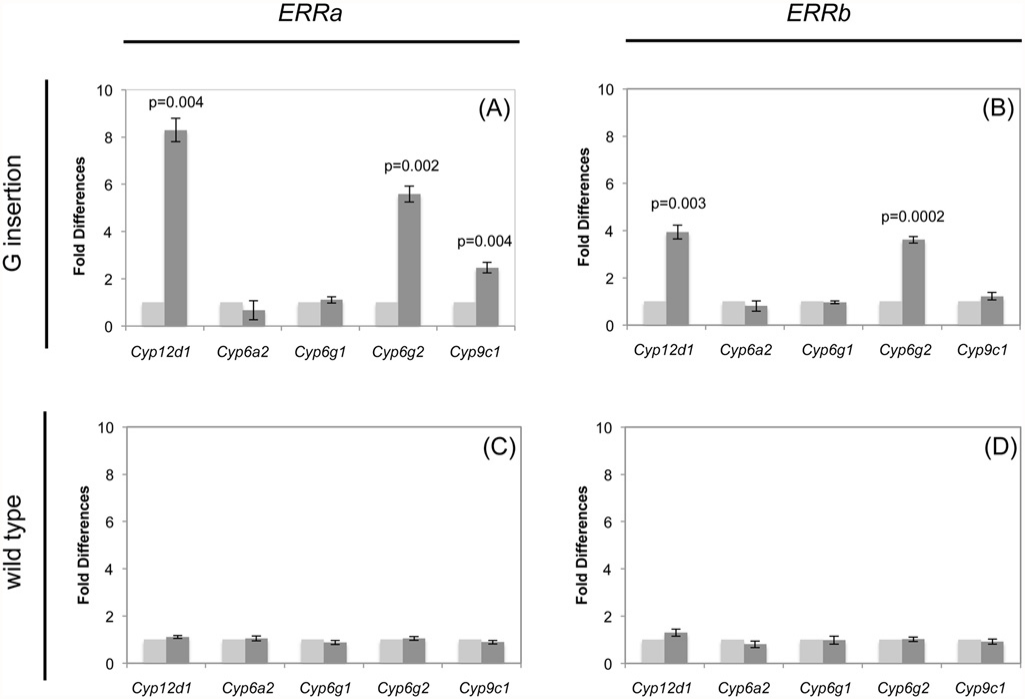
Expression of P450s in transgenic flies. Expression of five P450 genes in *w* ^*1118*^ transgenic flies expressing *ERRa* or *ERRb* cloned from *91-R* (G insertion) or *Canton-S* (wt) strains not crossed with a driver (light gray) or the progeny of a cross with the driver strain (darker gray). (A) *ERRa* from *91-R* resulted in three P450s being significantly over-transcribed after a Bonferonni correction. (B) *ERRb* from *91-R* resulted in two P450s being significantly over-transcribed after a Bonferonni correction. (C, D) Respectively *ERRa* and *ERRb* from *Canton-S* did not result in any of the P450s being significantly over-transcribed after a Bonferonni correction.

Both the *ERRa* and *ERRb* cDNAs, from *91-R*, contained the G codon insertion and caused a constitutive 3.5- to 8-fold over-expression of multiple P450 genes when expressed in a transgenic insect. In the transgenic flies expressing the *ERRa* cDNA from *91-R*, transcriptional levels of *Cyp12d1*, *Cyp6g2* and *Cyp9c1* were significantly increased (Fig. 2A). In transgenic flies, expression of the *ERRb* cDNA from *91-R*, significantly increased expression levels of *Cyp12d1* and *Cyp6g2*, but not *Cyp9c1* (Fig. 2B). However, the *ERRa* and *ERRb* transcripts from *Canton-S* (wt) that do not have the G codon insertion did not cause significant increases in P450 expression in any of the P450s tested when expressed in transgenic insects (Fig. 2C and 2D; *Cyp12d1* was not significantly over-transcribed after a Bonferroni correction in Fig. 2D).

### Analysis of *ERR* gene across *D. melanogaster* populations

**Fig 3:**
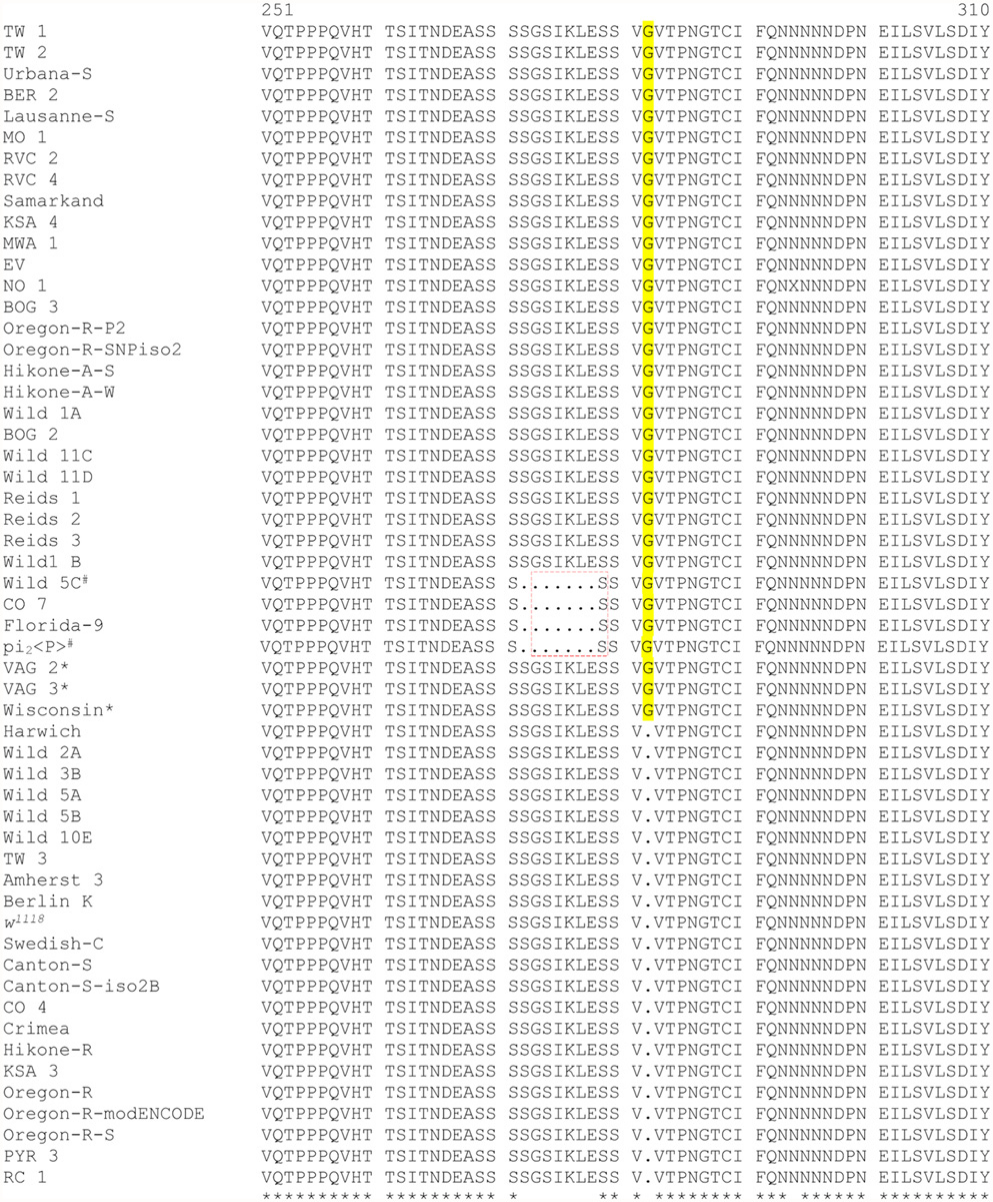
Comparison of *ERR* genomic sequences. Sequencing of *ERR*, corresponding to the amino acid range 251 to 310, revealed three *ERR* variants across 57 tested *Drosophila melanogaster* strains for alleles: (1) without the G insertion, (2) with the G insertion, and (3) with a deletion of seven codons plus the G insertion. Additionally, strains heterozygous for G insertion are designated with # and compound heterozygous (del+G / wt+G) strains are shown with *.

In order to determine the frequency of the G insertion allele across different *D. melanogaster* populations, a total of 57 strains (including *91-R* and *91-C*) were genotyped for a portion of the LBD of *ERR*, which was 160 amino acids in length. Our analysis revealed that the insertion of the G codon is a common event, occurring in 61.4% (35/57) of the *D. melanogaster* strains; with 54.4% (31/57) of the strains showing this allele in homozygosity and 7% (4/57) of them showing the allele in heterozygosity. In four strains (*CO 7*, *Florida-9*, *pi*_*2*_ and *Wild 5C)*, we found a third allele that had a 21 nucleotide deletion in combination with the G codon insertion that resulted a deletion of seven amino acids upstream of the insertion. This third combined allele (deletion + G) was present in homozygosity in 3.5% (2/57) of the strains tested. Strains *pi*_*2*_ and *Wild 5C* were heterozygous for del+G and wt+G (Fig. 3).

### The structural analyses of ERRa and ERRb proteins

Using NCBI PSI-BLAST and DELTA-BLAST, we were able to predict both the putative DNA binding domain (DBD) and LBD for the ERRa and ERRb proteins. For ERRa, the predicted zinc finger DBD occurred at the amino acid sequences from 109 to 205 in both *Canton-S* and *91-R* proteins. Additionally, the amino acid sequences from 225 to 482 were predicted to be the LBD, for both the *Canton-S* and *91-R* ERRa proteins. For ERRb, the DBD domain was predicted to be from amino acid 121 to 217 for both the *Canton-S* and *91-R* proteins. The LBD domain, however, was predicted to be from amino acid 237 to 493 for the *Canton-S* protein and from 237 to 494 for the *91-R* protein; the G insertion for *91-R* ERR was near the amino-end of the LBD domain (270 for ERRa and 282 for ERRb).

**Fig 4:**
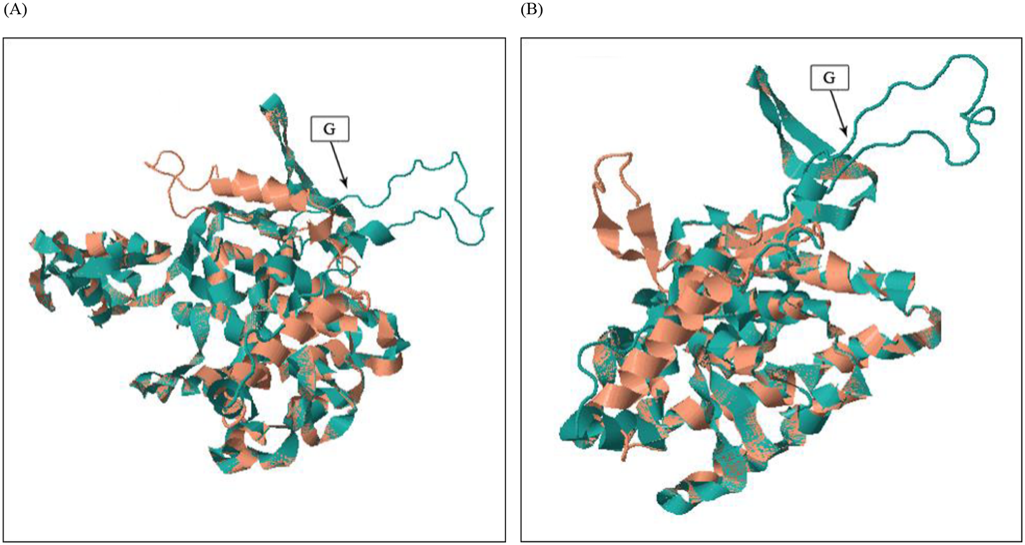
Protein structure comparisons. Predicted structures of ERRa and ERRb proteins superimposed between wt and G insertion. (A) Superimposed ERRa structures. (B) Superimposed ERRb structures. The orange color represents the wt ERR and green color represents ERR with a G insertion. The arrow points to the G insertion. The protein structure and Protein Data Bank (PDB) files were generated by using Swiss model First Approach mode (http://swissmodel.expasy.org/workspace/). The PDB files were used as input at http://mspc.bii.a-star.edu.sg/minhn/click.html to generate the superimposed structure images [22].

Pairwise structure alignment using the server on http://ekhidna.biocenter.helsinki.fi/dali_lite/start [25] indicates that the G insertion has caused structurally misalignment of about 40 amino acids at the amino-end of the insertion and about 12 amino acids at the carboxyl end of insertion in both ERRa and ERRb (S2 Fig. and S3 Fig.). The effect of this shift in the secondary structure was observed in the tertiary structure model generated by SWISS Model automated mode and superimposed by CLICK [22]. Superimposing of the tertiary structures of the wt ERRa and G ERRa revealed two loops not overlapping due to the G insertion (Fig. 4A). Similarly, unmatched loops were likewise observed when wt ERRb and G ERRb tertiary structures were superimposed (Fig. 4B).

### Analysis of ERR mutation across *Drosophila* species

**Fig 5:**
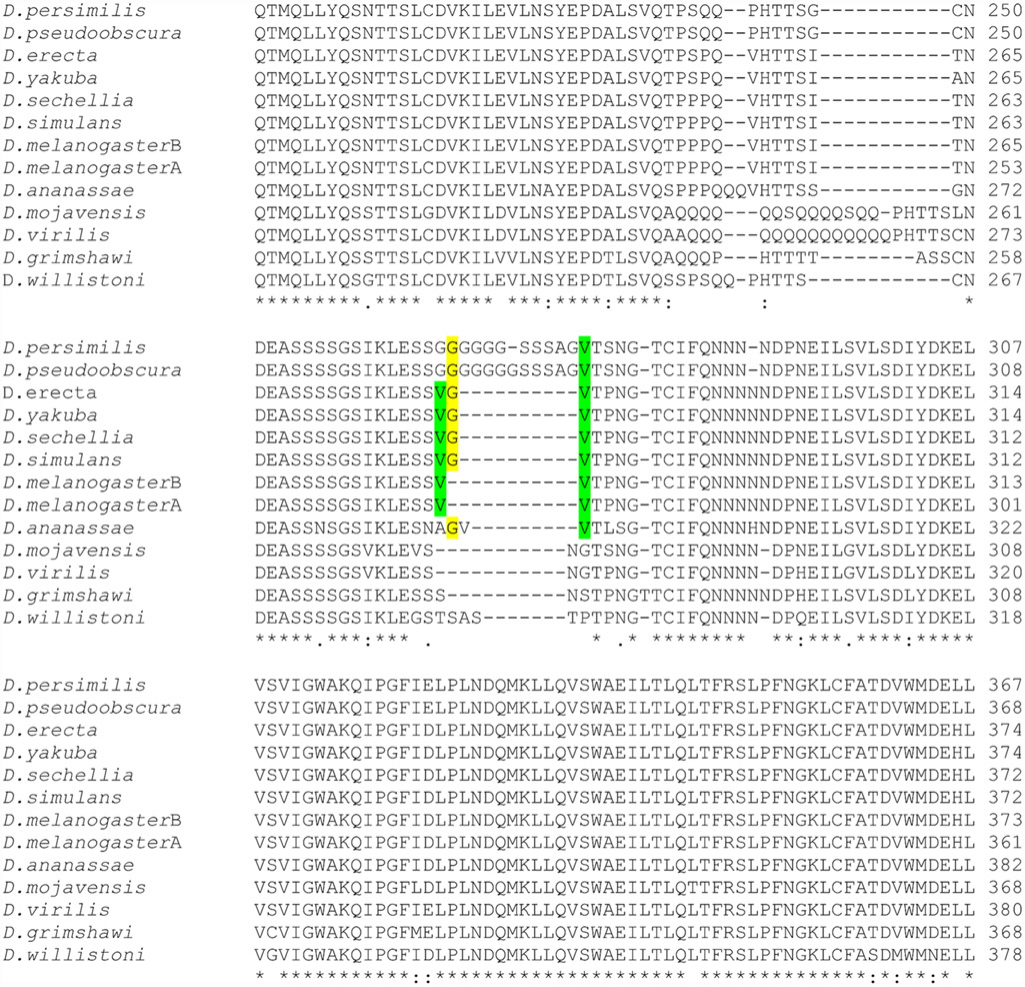
Clustal Omega Alignment of ERR. The ligand binding domain region of *Drosophila* species were compared. The sequences were downloaded from Hierarchical Catalog of Orthologs. Only the wt allele was included for *Drosophila melanogaster*.

**Fig 6:**
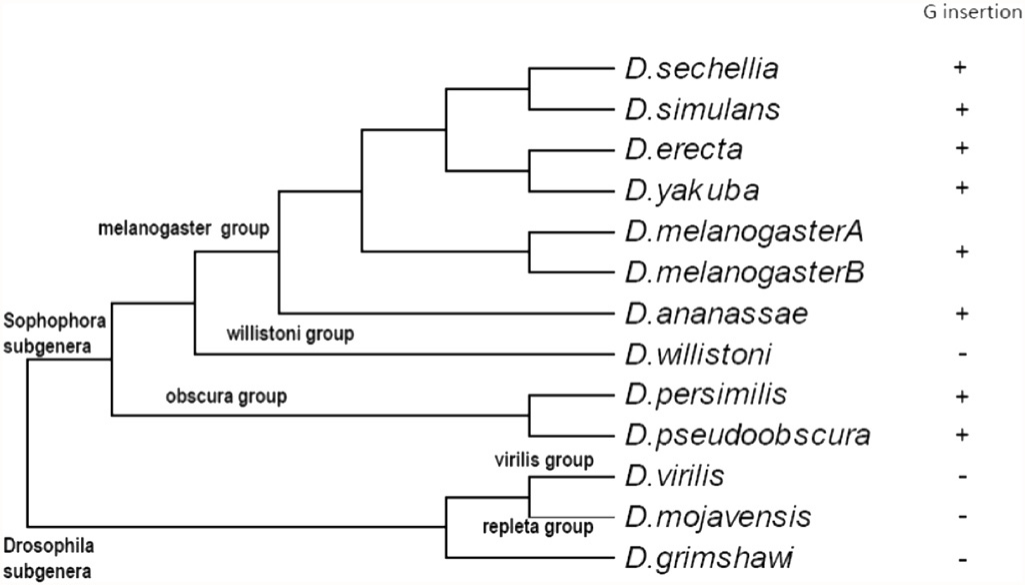
Molecular Phylogenetic analysis. The evolutionary history was inferred by using the Maximum Likelihood method based on the JTT matrix-based model [23]. The tree with the highest log likelihood (-2145.5939) is shown. Initial tree(s) for the heuristic search were obtained automatically by applying Neighbor-Join and BioNJ algorithms to a matrix of pairwise distances estimated using a JTT model, and then selecting the topology with superior log likelihood value. The tree is drawn to scale, with branch lengths measured in the number of substitutions per site. All positions containing gaps and missing data were eliminated. There were a total of 455 positions in the final dataset. Evolutionary analyses were conducted in MEGA5.2 [24].

To investigate if the G insertion was present in other *Drosophila* species, we made comparisons among the twelve available *Drosophila* species ERR sequences. The region in ERR, where the G insertion occurred in *D. melanogaster*, appears to be highly variable across the *Drosophila* species we investigated (Fig. 5). Additionally, insertion of the G codon occurs in multiple species across the Sophophora subgenera (Fig. 6).

## Discussion

The NR superfamily of genes codes for ligand-inducible transcription factors, in which are important for signaling pathways and coordination of the transcriptional response to specific ligands. Most NR proteins share a similar structure being composed of several modular domains, which are differentially conserved, including a highly conserved DBD, a variable hinge region and a less conserved LBD. The ERRs are a subfamily of the NR for which ligands have yet to be identified (so called orphan receptors). In humans, there are three *ERR*s (*α*, *β* and *γ*) that were identified through homology searches for genes coding for proteins related to estrogen receptors (*ERs*). In *Drosophila*, there is only a single *ERR* gene [26], which is orthologous to human *ERRγ*, and there is no *Drosophila* orthologous for human *ER*s. This finding suggests a common ancestor but the phylogeny of these genes remains controversial [27].

Studies, mainly in mammalians, have identified common mechanisms and divergent functions of ERRs [28]. To date, no natural ligand for ERRs has been described and the research approaches focus mostly on the physiological process under transcriptional control of ERRs. Recent research provides evidence that ERRs are master regulators of the mitochondrial biogenesis and function [29]. The fact that mammalian ERRs are expressed in tissues with high metabolic needs gave the first clues in determining the role of the ERRs in the regulation of energy metabolism [30]. In *Drosophila*, a recent study demonstrated the role of ERR in carbohydrate metabolism during larva stages [31] and a very recent paper shows an emerging role of *ERR* in hypoxic networks [32].

Here we described that an amino acid insertion in the hinge close to the 5’end of LBD of both *Drosophila* ERRa and ERRb in the *91-R* resistant strain and that this amino acid insertion caused constitutive over-expression of multiple cytochrome P450s when transformed into the *w* ^*1118*^ strain. This represents, to the authors’ knowledge, the first reported study of a naturally-occurring allele in *ERR* influencing the expression of the three xenobiotic responsive P450s, *Cyp12d1*, *Cyp6g2* and *Cyp9c1*, in adult *Drosophila*. The G insertion allele was also common across *D. melanogaster* strains as well as other *Drosophila* species. The G insertion and flanking deletion alleles were also observed in some strains of *D. melanogaster*. Previous analyses of the expression of several P450s in the larva of the *ERR* double mutant showed that *Cyp6a8* and *Cyp9c1* are the most up-regulated P450 genes [31].

Interestingly, *Cyp6g2* over-expression has been shown to be induced by nitenpyram and diazinon treatment [33]. *Cyp12d1* over-expression has been shown to be induced by DDT [18,33], dicyclanil [33], phenobarbital, hydrogen peroxide, heat (37°C) and starvation [19]. To date, and to the authors’ knowledge, there are no existing reports demonstrating any xenobiotics causing differential transcription of *Cyp9c1*. These results indicate that expression of at least two of these aforementioned P450s, *Cyp6g2* and *Cyp12d1*, are responsive to environmental or xenobiotic stresses or both.

The position of the G insertion is at amino acid 282 relative to ERRb, in the hinge close to the 5’end of LBD. An engineered triple mutation, Y295A/T333I/Y365L, in the ERR binding domain enabled the binding of a known ERR inverse agonists and suppressed the transcriptional activity of the receptor [34]. It is possible that the insertion of G close to this general region may alter the affinity of ERR to putative ligands and/or may make the DNA binding domain more accessible; a hypothesis that remains to be tested.

It has previously been shown that ERR is able to interact with either single or repeated response elements *in vitro* [34]. Our *in silico* search within 10 kb of 5 prime region (10 Kb upstream of the start codon) of the over-expressed P450s, *Cyp12d1*, *Cyp6g2* and *Cyp9c1*, revealed a putative ERR binding motif for all three genes that is conserved across the 12 *Drosophila* species with known genome (S2 Table). In contrast, the same approach was used in the search of the 10 kb of 5 prime regions of the non over-expressed P450s tested, *Cyp6g1* and *Cyp6a2* and, although several putative ERR binding sites were predicted none of them was conserved across the *Drosophila* species.

As a nuclear receptor protein, ERR may exert its effect through multiple regulatory pathways. It has been shown that the nuclear receptor, DHR96, which is orthologous to two human NR, Steroid and Xenobiotic Receptor (SXR) and Constitutive Androstane Receptor (CAR), controls only a small portion of the phenobarbital (PB) response [35]. The Nrf2/Keap1 pathway is also thought to regulate xenobiotic responsive genes [15]. Recent results from the same research group show that the constitutive activation of the Nrf2/Keap1 pathway can contribute to the overexpression of detoxifying genes in resistant strains but the mechanism(s) remain unknown [36]. The regulation of NRF2 pathway by ERs and ERRs is well documented in mammalians [37–40]. Specifically, it has been shown that ERRβ (particularly the short isoform SFhERRβ) interacts with NRF2 and inhibits its transcriptional activity [41].

Our results show that the pattern of expression of both *ERR* isoforms is different between *Drosophila* strains (Table 1), particularly the levels of *ERRa* expression in the *91-R* resistant strain, which is more than two times that of the level of expression in the susceptible *Canton-S* strain. It has been described an association between the constitutive over-expression of *Cyp6a2* with an allele containing an intact Nrf2/Maf-binding-site [16]. We were not able to identify a highly conserved Nrf2 binding site close to the locations of the putative ERR binding site described among 12 *Drosophila* species (S2 Table). Finally, it has been postulated that mutations in repressor gene(s) on the third chromosome may be one reason for the constitutive over-expression of *Cyp6a2* and *Cyp6a8* in resistant *Drosophila* strains [42]. Similar to the *dNrf2* and *dKeap1* genes, *ERR* is also on the third chromosome. It remains to be determined, however, if ERR is a regulator of Nrf2/Keap1pathway. Further experimentation will need to be performed to test this hypothesis.

Future studies, using antibodies, might be able to determine protein levels of ERR between the wt and G insertion EER alleles. We speculate that the level of ERR protein would not be different between wt and G insertion ERRs. The 3D protein modeling (Fig. 4, S1 Fig. and S2 Fig.) indicates that the G insertion causes tertiary structural changes to the ERR proteins. These changes might have increased the ERRs DNA binding to these three CYPs transcription factor binding motifs after binding its putative ligand. As of now, no known physiological ligand has been identified to bind either mammalian or insect ERR proteins.

## Conclusion

In the present study, we observed that transgenic flies expressing an *ERR* gene with the amino acid G insertion in the resultant ERR protein caused the constitutive over-expression of the P450s, *Cyp12d1*, *Cyp6g2* and *Cyp9c1*, all known to be involved in xenobiotic responses. The mechanism of how this *ERR* G insertion influences *Cyp12d1*, *Cyp6g2* and *Cyp9c1* expression, potentially through the change of ERR protein structure, remains to be experimentally determined as does the role of native copies of this allele across *D. melanogaster* strains. The fact that the G insertion can be found across many other *Drosophila* species in the Sophophora subgenera suggests that either this insertion is very old evolutionally, or it has occurred independently multiple times. However, the precise role or selective advantage, if any, that this allele may play in populations of *D. melanogaster*, and other species of *Drosophila*, remains to be determined.

## Supporting Information

S1 Fig Glycine insertion in ERR. Relative position of G insertion in ERRa and ERRb in *91-R* amino acids sequences as compared to *Canton-S*.
(PDF)

S2 Fig Pairwise structure alignment for ERRa in wt and G insertion. The alignment was generated by using server on http://ekhidna.biocenter.helsinki.fi/dali_lite/start [25]. The PDB file for each ERR amino acid sequence was used as input files to generate these alignment figure. The PDB file for each ERR amino acid sequence was generated at SWISS-MODEL (http://swissmodel.expasy.org/; [21]). Notation: three-state secondary structure definitions by define secondary structure of proteins (DSSP) algorithm (reduced to H = helix, E = sheet, L = coil) are shown above the amino acid sequence. Structurally equivalent residues are in uppercase, structurally non-equivalent residues (e.g. in loops) are in lowercase. Amino acid identities are marked by vertical bars (ident). G insertion is highlighted in yellow. Pairwise structure alignment for ERRa between wt and G.
(PDF)

S3 Fig Pairwise structure alignment for ERRb in wt and G insertion. The alignment was generated by using server on http://ekhidna.biocenter.helsinki.fi/dali_lite/start [25]. The Protein Data Bank (PDB) file for each ERR amino acid sequence was used as input files to generate these alignment figure. The PDB file for each ERR amino acid sequence was generated at SWISS-MODEL (http://swissmodel.expasy.org/; [21]). Notation: three-state secondary structure definitions by DSSP (reduced to H = helix, E = sheet, L = coil) are shown above the amino acid sequence. Structurally equivalent residues are in uppercase, structurally non-equivalent residues (e.g. in loops) are in lowercase. Amino acid identities are marked by vertical bars (ident). G insertion is highlighted in yellow. Pairwise structure alignment for ERRb between wt and G.
(PDF)

S1 Table Primer Sequences for ERR cloning, sequencing, qRT-PCR and genomic amplification of the ligand binding domain (LBD) of *ERR*. (PDF)

S2 Table Predicted ERR binding sites identified within tested P450s. Different programs, JASPAR [43], PROMO [44], Genome Surveyor [45], Math (TRANSFAC) (http://www.bioinfo.de/isb/gcb01/poster/index.html) and MEME [46], were used as motif discovery tools to analyze the 10 kb upstream region of a set of P450s. Conservation analyses of the putative ERR binding site regions in 12 *Drosophila* species were performed through UCSC Genome Browser [47]. Coordinates for the putative ERR binding sites are referred to *Drosophila* genome R6.01. The logos were generated using WebLogo software [48]. The logo height of the letter indicates the probability of appearing at the position in the motifs. A consensus ERR binding motif was generated using the sequences of the predicted sites for the up-regulated P450s in the 12 *Drosophila* species.
(PDF)

## References

[1] ffrench-Constant RH, Daborn PJ, Le Goff G. The genetics and genomics of insecticide resistance. Trends Genet. 2004;20(3):163–70. 10.1016/j.tig.2004.01.003 15036810

[2] Li XC, Schuler MA, Berenbaum MR. Molecular mechanisms of metabolic resistance to synthetic and natural xenobiotics. Annu Rev Entomol. 2007;52:231–53. 10.1146/annurev.ento.51.110104.151104 16925478

[3] Oakeshott JG, Horne I, Sutherland TD, Russell RJ. The genomics of insecticide resistance. Genome Biol. 2003;4(1). 10.1186/Gb-2003.4.1.202 12540295 PMC151287

[4] Amichot M, Tares S, Brun-Barale A, Arthaud L, Bride JM, Berge JB. Point mutations associated with insecticide resistance in the *Drosophila* cytochrome P450 *Cyp6a2* enable DDT metabolism. Eur J Biochem. 2004;271(7):1250–7. 10.1111/j.1432-1033.2004.04025.x 15030474

[5] Le Goff G, Boundy S, Daborn PJ, Yen JL, Sofer L, Lind R, et al Microarray analysis of cytochrome P450 mediated insecticide resistance in *Drosophila* . Insect Biochem Mol Biol. 2003;33(7):701–8. 10.1016/S0965-1748(03)00064-X 12826097

[6] Pedra JH, McIntyre LM, Scharf ME, Pittendrigh BR. Genome-wide transcription profile of field- and laboratory-selected dichlorodiphenyltrichloroethane (DDT)-resistant *Drosophila* . Proc Natl Acad Sci U S A. 2004;101(18):7034–9. 10.1073/pnas.0400580101 15118106 PMC406461

[7] McKenzie JA, Batterham P. Predicting insecticide resistance: mutagenesis, selection and response. Phil Trans R Soc Lond B. 1998;353(1376):1729–34. 10.1098/rstb.1998.0325 10021773 PMC1692398

[8] Catania F, Kauer MO, Daborn PJ, Yen JL, Ffrench-Constant RH, Schlotterer C. World-wide survey of an Accord insertion and its association with DDT resistance in *Drosophila melanogaster* . Mol Ecol. 2004;13(8):2491–504. 10.1111/j.1365-294X.2004.02263.x.9 15245421

[9] Kuruganti S, Lam V, Zhou X, Bennett G, Pittendrigh BR, Ganguly R. High expression of *Cyp6g1*, a cytochrome P450 gene, does not necessarily confer DDT resistance in *Drosophila melanogaster* . Gene. 2007;388(1–2):43–53. 10.1016/j.gene.2006.09.019 17134855

[10] Festucci-Buselli RA, Carvalho-Dias AS, de Oliveira-Andrade M, Caixeta-Nunes C, Li HM, Stuart JJ, et al Expression of *Cyp6g1* and *Cyp12d1* in DDT resistant and susceptible strains of *Drosophila melanogaster* . Insect Mol Biol. 2005;14(1):69–77. 10.1111/j.1365-2583.2005.00532.x 15663776

[11] Giraudo M, Unnithan GC, Le Goff G, Feyereisen R. Regulation of cytochrome P450 expression in *Drosophila*: Genomic insights. Pestic Biochem Physiol. 2010;97(2):115–22. 10.1016/j.pestbp.2009.06.009 20582327 PMC2890303

[12] Qiu X, Sun W, McDonnell CM, Li-Byarlay H, Steele LD, Wu J, et al Genome-wide analysis of genes associated with moderate and high DDT resistance in *Drosophila melanogaster* . Pest Manag Sci. 2013;69:930–7. 10.1002/ps.3454 23371854

[13] Strycharz JP, Lao A, Li HM, Qiu XH, Lee SH, Sun WL, et al Resistance in the highly DDT-resistant 91-R strain of *Drosophila melanogaster* involves decreased penetration, increased metabolism, and direct excretion. Pestic Biochem Physiol. 2013;107(2):207–17. 10.1016/j.pestbp.2013.06.010

[14] Plapp FW. The Genetic-Basis of Insecticide Resistance in the Housefly—Evidence That a Single Locus Plays a Major Role in Metabolic Resistance to Insecticides. Pestic Biochem Physiol. 1984;22(2):194–201. 10.1016/0048-3575(84)90089-0 15.

[15] Misra JR, Horner MA, Lam G, Thummel CS. Transcriptional regulation of xenobiotic detoxification in *Drosophila* . Genes Dev. 2011;25(17):1796–806. 10.1101/Gad.17280911 21896655 PMC3175716

[16] Wan H, Liu Y, Li M, Zhu S, Li X, Pittendrigh BR, et al Nrf2/Maf-binding-site-containing functional *Cyp6a2* allele is associated with DDT resistance in *Drosophila melanogaster* . Pest Manag Sci. 2014;70(7):1048–58. 10.1002/ps.3645 24038867

[17] Dvorak Z, Pavek P. Regulation of drug-metabolizing cytochrome P450 enzymes by glucocorticoids. Drug Metab Rev. 2010;42(4):621–35. 10.3109/03602532.2010.484462 20482443

[18] Brandt A, Scharf M, Pedra JH, Holmes G, Dean A, Kreitman M, et al Differential expression and induction of two *Drosophila* cytochrome P450 genes near the *Rst(2)DDT* locus. Insect Mol Biol. 2002;11(4):337–41. 12144699 10.1046/j.1365-2583.2002.00344.x

[19] McDonnell CM, King D, Comeron JM, Li HM, Sun WL, Berenbaum MR, et al Evolutionary Toxicogenomics: Diversification of the *Cyp12d1* and *Cyp12d3* Genes in *Drosophila* Species. J Mol Evol. 2012;74(5–6):281–96. 10.1007/s00239-012-9506-3 22811321

[20] Pfaffl MW, Horgan GW, Dempfle L. Relative expression software tool (REST) for group-wise comparison and statistical analysis of relative expression results in real-time PCR. Nucleic Acids Res. 2002;30(9):e36 11972351 10.1093/nar/30.9.e36 PMC113859

[21] Arnold K, Bordoli L, Kopp J, Schwede T. The SWISS-MODEL workspace: a web-based environment for protein structure homology modelling. Bioinformatics. 2006;22(2):195–201. 10.1093/bioinformatics/bti770 16301204

[22] Nguyen MN, Tan KP, Madhusudhan MS. CLICK—topology-independent comparison of biomolecular 3D structures. Nucleic Acids Res. 2011;39(Web Server issue):W24–28. 10.1093/nar/gkr393 21602266 PMC3125785

[23] Jones DT, Taylor WR, Thornton JM. The rapid generation of mutation data matrices from protein sequences. Comput Appl Biosci. 1992;8(3):275–282. 1633570 10.1093/bioinformatics/8.3.275

[24] Tamura K, Peterson D, Peterson N, Stecher G, Nei M, Kumar S. MEGA5: molecular evolutionary genetics analysis using maximum likelihood, evolutionary distance, and maximum parsimony methods. Mol Biol Evol. 2011;28(10):2731–2739. 10.1093/molbev/msr121 21546353 PMC3203626

[25] Holm L, Park J. DaliLite workbench for protein structure comparison. Bioinformatics. 2000;16(6):566–567. 10980157 10.1093/bioinformatics/16.6.566

[26] Sullivan AA, Thummel CS. Temporal profiles of nuclear receptor gene expression reveal coordinate transcriptional responses during *Drosophila* development. Mol Endocrinol. 2003;17(11):2125–2137. 10.1210/Me.2002-0430 12881508

[27] Bardet PL, Laudet V, Vanacker JM. Studying non-mammalian models? Not a fool’s ERRand! Trends Endocrinol Metab. 2006;17(4):166–1671. 10.1016/j.tem.2006.03.005 16580224 PMC1868322

[28] Tremblay AM, Giguere V. The NR3B subgroup: an ovERRview. Nucl Recept Signal. 2007;5:e009 10.1621/nrs.05009 18174917 PMC2121319

[29] Eichner LJ, Giguere V. Estrogen related receptors (ERRs): A new dawn in transcriptional control of mitochondrial gene networks. Mitochondrion. 2011;11(4):544–52. 10.1016/j.mito.2011.03.121 21497207

[30] Giguere V. Transcriptional Control of Energy Homeostasis by the Estrogen-Related Receptors. Endocr Rev. 2008;29(6):677–96. 10.1210/Er.2008-0017 18664618

[31] Tennessen JM, Baker KD, Lam G, Evans J, Thummel CS. The *Drosophila* Estrogen-Related Receptor Directs a Metabolic Switch that Supports Developmental Growth. Cell Metabolism. 2011;13(2):139–48. 10.1016/j.cmet.2011.01.005 21284981 PMC3072597

[32] Li Y, Padmanabha D, Gentile LB, Dumur CI, Beckstead RB, Baker KD. HIF- and Non-HIF-Regulated Hypoxic Responses Require the Estrogen-Related Receptor in *Drosophila melanogaster* . PLoS Genet. 2013;9(1). 10.1371/journal.pgen.1003230 PMC3561118 23382692

[33] Daborn PJ, Lumb C, Boey A, Wong W, Ffrench-Constant RH, Batterham P. Evaluating the insecticide resistance potential of eight *Drosophila melanogaster* cytochrome P450 genes by transgenic over-expression. Insect Biochem Mol Biol. 2007;37(5):512–9. 10.1016/j.ibmb.2007.02.008 17456446

[34] Ostberg T, Jacobsson M, Attersand A, Mata de Urquiza A, Jendeberg L. A triple mutant of the *Drosophila ERR* confers ligand-induced suppression of activity. Biochemistry. 2003;42(21):6427–35. 10.1021/bi027279b 12767224

[35] King-Jones K, Horner MA, Lam G, Thummel CS. The DHR96 nuclear receptor regulates xenobiotic responses in *Drosophila* . Cell Metab. 2006;4(1):37–48. 10.1016/j.cmet.2006.06.006.36 16814731

[36] Misra JR, Lam G, Thummel CS. Constitutive activation of the Nrf2/Keap1 pathway in insecticide-resistant strains of *Drosophila* . Insect Biochem Mol Biol. 2013;43(12):1116–24. 10.1016/j.ibmb.2013.09.005 24099738 PMC3852162

[37] Ariazi EA, Clark GM, Mertz JE. Estrogen-related receptor alpha and estrogen-related receptor gamma associate with unfavorable and favorable biomarkers, respectively, in human breast cancer. Cancer Res. 2002;62(22):6510–8. 12438245

[38] Ansell PJ, Lo SC, Newton LG, Espinosa-Nicholas C, Zhang DD, Liu JH, et al Repression of cancer protective genes by 17beta-estradiol: ligand-dependent interaction between human Nrf2 and estrogen receptor alpha. Mol Cell Endocrinol. 2005;243(1–2):27–34. 10.1016/j.mce.2005.08.002 16198475

[39] Kensler TW, Wakabayashi N, Biswal S. Cell survival responses to environmental stresses via the Keap1-Nrf2-ARE pathway. Annu Rev Pharmacol Toxicol. 2007;47:89–116. 10.1146/annurev.pharmtox.46.120604.141046 16968214

[40] Niture SK, Khatri R, Jaiswal AK. Regulation of Nrf2—an update. Free Radical Biol Med. 2014;66:36–44. 10.1016/j.freeradbiomed.2013.02.008 23434765 PMC3773280

[41] Zhou W, Lo SC, Liu JH, Hannink M, Lubahn DB. ERR beta: A potent inhibitor of Nrf2 transcriptional activity. Mol Cell Endocrinol. 2007;278(1–2):52–62. 10.1016/j.mce.2007.08.011 17920186

[42] Maitra S, Dombrowski SM, Basu M, Raustol O, Waters LC, Ganguly R. Factors on the third chromosome affect the level of *Cyp6a2* and *Cyp6a8* expression in *Drosophila melanogaster* . Gene. 2000;248(1–2):147–56. 10.1016/S0378-1119(00)00129-3 10806360

[43] Mathelier A, Zhao X, Zhang AW, Parcy F, Worsley-Hunt R, Arenillas DJ, et al JASPAR 2014: an extensively expanded and updated open-access database of transcription factor binding profiles. Nucleic Acids Res. 2014;42(Database issue):D142–7. 10.1093/nar/gkt997 24194598 PMC3965086

[44] Messeguer X, Escudero R, Farre D, Nunez O, Martinez J, Alba M. PROMO: detection of known transcription regulatory elements using species-tailored searches. Bioinformatics. 2002;18(2):333–4. 10.1093/bioinformatics/18.2.333 11847087

[45] Kazemian M, Brodsky MH, Sinha S. Genome surveyor 2.0: cis-regulatory analysis in *Drosophila* . Nucleic Acids Res. 2011;39:W79–W85. 10.1093/Nar/Gkr291 21593125 PMC3125742

[46] Bailey TL, Boden M, Buske FA, Frith M, Grant CE, Clementi L, et al MEME SUITE: tools for motif discovery and searching. Nucleic Acids Res. 2009;37:W202–W8. 10.1093/Nar/Gkp335 19458158 PMC2703892

[47] Karolchik D, Barber GP, Casper J, Clawson H, Cline MS, Diekhans M, et al The UCSC Genome Browser database: 2014 update. Nucleic Acids Res. 2014;42(D1):D764–D70. 10.1093/Nar/Gkt1168 24270787 PMC3964947

[48] Crooks GE, Hon G, Chandonia JM, Brenner SE. WebLogo: A sequence logo generator. Genome Res. 2004;14(6):1188–90. 10.1101/Gr.849004 15173120 PMC419797

