# Supplementary material for: A Glycine Insertion in the Estrogen-Related Receptor (ERR) Is Associated with Enhanced Expression of Three Cytochrome P450 Genes in Transgenic Drosophila melanogaster
Source: PLoS One. 2015 Mar 11;10(3):e0118779. doi: 10.1371/journal.pone.0118779 (PMC4356566; doi:10.1371/journal.pone.0118779)
Supplement: S1 Fig — Relative position of G insertion in ERRa and ERRb in 91-R amino acids sequences as compared to Canton-S. (PDF) [file pone.0118779.s001.pdf]

```
            201                                                250
ERRa_91R    RVRGGRQKYR RNPVSNSYQT MQLLYQSNTT SLCDVKILEV LNSYEPDALS
ERRa_CS     RVRGGRQKYR RNPVSNSYQT MQLLYQSNTT SLCDVKILEV LNSYEPDALS
ERRb_91R    RVRGGRQKYR RNPVSNSYQT MQLLYQSNTT SLCDVKILEV LNSYEPDALS
ERRb_CS     RVRGGRQKYR RNPVSNSYQT MQLLYQSNTT SLCDVKILEV LNSYEPDALS

            251                                                300
ERRa_91R    VQTPPPQVHT TSITNDEASS SSGSIKLESS VGVTPNGTCI FQNNNNNDPN
ERRa_CS     VQTPPPQVHT TSITNDEASS SSGSIKLESS V.VTPNGTCI FQNNNNNDPN
ERRb_91R    VQTPPPQVHT TSITNDEASS SSGSIKLESS VGVTPNGTCI FQNNNNNDPN
ERRb_CS     VQTPPPQVHT TSITNDEASS SSGSIKLESS V.VTPNGTCI FQNNNNNDPN

            301                                                350
ERRa_91R    EILSVLSDIY DKELVSVIGW AKQIPGFIDL PLNDQMKLLQ VSWAEILTLQ
ERRa_CS     EILSVLSDIY DKELVSVIGW AKQIPGFIDL PLNDQMKLLQ VSWAEILTLQ
ERRb_91R    EILSVLSDIY DKELVSVIGW AKQIPGFIDL PLNDQMKLLQ VSWAEILTLQ
ERRb_CS     EILSVLSDIY DKELVSVIGW AKQIPGFIDL PLNDQMKLLQ VSWAEILTLQ

            351                                                400
ERRa_91R    LTFRSLPFNG KLCFATDVWM DEHLAKECGY TEFYYHCVQI AQRMERISPR
ERRa_CS     LTFRSLPFNG KLCFATDVWM DEHLAKECGY TEFYYHCVQI AQRMERISPR
ERRb_91R    LTFRSLPFNG KLCFATDVWM DEHLAKECGY TEFYYHCVQI AQRMERISPR
ERRb_CS     LTFRSLPFNG KLCFATDVWM DEHLAKECGY TEFYYHCVQI AQRMERISPR

            401                                                450
ERRa_91R    REEYYLLKAL LLANCDILLD DQSSLRAFRD TILNSLNDVV YLLRHSSAVS
ERRa_CS     REEYYLLKAL LLANCDILLD DQSSLRAFRD TILNSLNDVV YLLRHSSAVS
ERRb_91R    REEYYLLKAL LLANCDILLD DQSSLRAFRD TILNSLNDVV YLLRHSSAVS
ERRb_CS     REEYYLLKAL LLANCDILLD DQSSLRAFRD TILNSLNDVV YLLRHSSAVS

            451                                            497
ERRa_91R    HQQQLLLLLP SLRQADDILR RFWRGIARDE VITMKKLFLE MLEPLAR
ERRa_CS     HQQQLLLLLP SLRQADDILR RFWRGIARDE VITMKKLFLE MLEPLAR
ERRb_91R    HQQQLLLLLP SLRQADDILR RFWRGIARDE VITMKKLFLE MLEPLAR
ERRb_CS     HQQQLLLLLP SLRQADDILR RFWRGIARDE VITMKKLFLE MLEPLAR
```
