# Supplementary material for: A Glycine Insertion in the Estrogen-Related Receptor (ERR) Is Associated with Enhanced Expression of Three Cytochrome P450 Genes in Transgenic Drosophila melanogaster
Source: PLoS One. 2015 Mar 11;10(3):e0118779. doi: 10.1371/journal.pone.0118779 (PMC4356566; doi:10.1371/journal.pone.0118779)
Supplement: S2 Fig — The alignment was generated by using server on http://ekhidna.biocenter.helsinki.fi/dali_lite/start [25]. The PDB file for each ERR amino acid sequence was used as input files to generate these alignment figure. The PDB file for each ERR amino acid sequence was generated at SWISS-MODEL (http://swissmodel.expasy.org/; [21]). Notation: three-state secondary structure definitions by define secondary structure of proteins (DSSP) algorithm (reduced to H = helix, E = sheet, L = coil) are shown above the amino acid sequence. Structurally equivalent residues are in uppercase, structurally non-equivalent residues (e.g. in loops) are in lowercase. Amino acid identities are marked by vertical bars (ident). G insertion is highlighted in yellow. Pairwise structure alignment for ERRa between wt and G. (PDF) [file pone.0118779.s002.pdf]

```
DSSP  LLLLLLLLLLLLEELLEELLHHHHHHHHHHHHHLLLLLLLLLLLLLLLLLLLLLLHHHHH
wt    RLCLVCGDVASGFHYGVASCEACKAFFKRTIQGNIEYTCPANNECEINKRRRKACQACRF   60
ident ||||||||||||||||||||||||||||||||||||||||||||||||||||||||||||
G     RLCLVCGDVASGFHYGVASCEACKAFFKRTIQGNIEYTCPANNECEINKRRRKACQACRF   60
DSSP  LLLLLLLLLLLLEELLEELLHHHHHHHHHHHHHLLLLLLLLLLLLLLLLLLLLLLHHHHH

DSSP  HHHHHHLLLHHHLLLLLLLLLLLLLLLLLEEEELLLEEEEELLLLLLLLLLlllllllll
wt    QKCLLMGMLKEGVRLDRVRGGRQKYRRNPVSNSYQTMQLLYQSNTTSLCDVkilevlnsy  120
ident |||||||||||||||||||||||||||||||||||||||||||||||||||
G     QKCLLMGMLKEGVRLDRVRGGRQKYRRNPVSNSYQTMQLLYQSNTTSLCDV---------  111
DSSP  HHHHHHLLLHHHLLLLLLLLLLLLLLLLLEEEELLLEEEEELLLLLLLLLL---------

DSSP  lllllllllhhhhhhhhHHLLH--------------------------hhlLHHHHLLll
wt    epdalsvqtpppqvhttSITND--------------------------easSSSGSIKle  154
ident                                                    | |
G     ---------------kiLEVLNsyepdalsvqtpppqvhttsitndeasssSGSIKLE--  154
DSSP  ---------------llLLLLLllllllllllllllllllllllllhhhllLLHHHHH--

DSSP  lllhhhhHHHHhhhhhhHLLLL--LLLLHHHHHHHHHHHHHHHHHHLLLLLLLLHHHHHH
wt    ssvvtpnGTCIfqnnnnNDPNE--ILSVLSDIYDKELVSVIGWAKQIPGFIDLPLNDQMK  212
ident                  |      ||||||||||||||||||||||||||||||||||||
G     ssvgvtpNGTC-ifqnnNNNDPneILSVLSDIYDKELVSVIGWAKQIPGFIDLPLNDQMK  213
DSSP  llhhhllLLLL-llllhHHHHHhhLHHHHHHHHHHHHHHHHHHHHHLLLLLLLLHHHHHH

DSSP  HHHLLHHHHHHHHHHHHLLLLLLLLLLLLLLLLLHHHHLLLHHHHLLLLLLHHHHHHHHH
wt    LLQVSWAEILTLQLTFRSLPFNGKLCFATDVWMDEHLAKECGYTEFYYHCVQIAQRMERI  272
ident ||||||||||||||||||||||||||||||||||||||||||||||||||||||||||||
G     LLQVSWAEILTLQLTFRSLPFNGKLCFATDVWMDEHLAKECGYTEFYYHCVQIAQRMERI  273
DSSP  HHHLLHHHHHHHHHHHHLLLLLLLLLLLLLLLLLHHHHLLLHHHHLLLLLLHHHHHHHHH

DSSP  LLLHHHHHHHHHHHHLLLLLLLLHHHHHHHHHHHHHHHHHHHHHHLLLLLLLLHHHHHHL
wt    SPRREEYYLLKALLLANCDILLDDQSSLRAFRDTILNSLNDVVYLLRHSSAVSHQQQLLL  332
ident ||||||||||||||||||||||||||||||||||||||||||||||||||||||||||||
G     SPRREEYYLLKALLLANCDILLDDQSSLRAFRDTILNSLNDVVYLLRHSSAVSHQQQLLL  333
DSSP  LLLHHHHHHHHHHHHLLLLLLLLHHHHHHHHHHHHHHHHHHHHHHLLLLLLLLHHHHHHL

DSSP  HHHHHHHHHHHHHHHHLLLLLLLLLLLLLHHHHLL
wt    LLPSLRQADDILRRFWRGIARDEVITMKKLFLEML  367
ident |||||||||||||||||||||||||||||||||||
G     LLPSLRQADDILRRFWRGIARDEVITMKKLFLEML  368
DSSP  HHHHHHHHHHHHHHHHLLLLLLLLLLLLLHHHHLL
```
