# Supplementary material for: A Glycine Insertion in the Estrogen-Related Receptor (ERR) Is Associated with Enhanced Expression of Three Cytochrome P450 Genes in Transgenic Drosophila melanogaster
Source: PLoS One. 2015 Mar 11;10(3):e0118779. doi: 10.1371/journal.pone.0118779 (PMC4356566; doi:10.1371/journal.pone.0118779)
Supplement: S3 Fig — The alignment was generated by using server on http://ekhidna.biocenter.helsinki.fi/dali_lite/start [25]. The Protein Data Bank (PDB) file for each ERR amino acid sequence was used as input files to generate these alignment figure. The PDB file for each ERR amino acid sequence was generated at SWISS-MODEL (http://swissmodel.expasy.org/; [21]). Notation: three-state secondary structure definitions by DSSP (reduced to H = helix, E = sheet, L = coil) are shown above the amino acid sequence. Structurally equivalent residues are in uppercase, structurally non-equivalent residues (e.g. in loops) are in lowercase. Amino acid identities are marked by vertical bars (ident). G insertion is highlighted in yellow. Pairwise structure alignment for ERRb between wt and G. (PDF) [file pone.0118779.s003.pdf]

```
DSSP  LLLLLLLLLLLLEELLEELLHHHHHHHHHHHHLLLLLLLLLLLLLLLLLLLLLLLHHHHH
wt    RLCLVCGDVASGFHYGVASCEACKAFFKRTIQGNIEYTCPANNECEINKRRRKACQACRF   60
ident ||||||||||||||||||||||||||||||||||||||||||||||||||||||||||||
G     RLCLVCGDVASGFHYGVASCEACKAFFKRTIQGNIEYTCPANNECEINKRRRKACQACRF   60
DSSP  LLLLLLLLLLLLEELLEELLHHHHHHHHHHHHLLLLLLLLLLLLLLLLLLLLLLLHHHHH

DSSP  HHHHHLLLHHHLLLLLLLLLLLLLLLLLLEEEELLLEEEEELLLLLLLLLLEE--ellll
wt    QKCLLMGMLKEGVRLDRVRGGRQKYRRNPVSNSYQTMQLLYQSNTTSLCDVKI--levln  118
ident |||||||||||||||||||||||||||||||||||||||||||||||||||||
G     QKCLLMGMLKEGVRLDRVRGGRQKYRRNPVSNSYQTMQLLYQSNTTSLCDVKIlevlnsy  120
DSSP  HHHHHLLLHHHLLLLLLLLLLLLLLLLLLEEEELLLEEEEELLLLLLLLLLLLlllllll

DSSP  leeeellllllllleeelllllllleeelLHHHHLLlllllhhhhHHHHhhhhhhHLLLL--
wt    syepdalsvqtpppqvhttsitndeasSSSGSIKlessvvtpnGTCIfqnnnnNDPNE--  176
ident                            | |                       |
G     epdalsvqtpppqvhttsitndeasssSGSIKLE--ssvgvtpNGTC-ifqnnNNNDPne  177
DSSP  llllllllllllllllllllllhhhllLLHHHHH--llhhhllLLLL-llllhHHHHHhh

DSSP  LLLHHHHHHHHHLHHHHHHHHLLLLLLLLLLHHHHHHHLLHHHHHHHHHHHHHLLLLLLL
wt    ILSVLSDIYDKELVSVIGWAKQIPGFIDLPLNDQMKLLQVSWAEILTLQLTFRSLPFNGK  236
ident ||||||||||||||||||||||||||||||||||||||||||||||||||||||||||||
G     ILSVLSDIYDKELVSVIGWAKQIPGFIDLPLNDQMKLLQVSWAEILTLQLTFRSLPFNGK  237
DSSP  LHHHHHHHHHHHHHHHHHHHHLLLLLLLLHHHHHHHHHLLHHHHHHHHHHHHHLLLLLLL

DSSP  LLLLLLLLLLLHHHHLLLLHHHHLLLLLLHHHHHHHLLLHHHHHHHHHHHHHLLLLLLLL
wt    LCFATDVWMDEHLAKECGYTEFYYHCVQIAQRMERISPRREEYYLLKALLLANCDILLDD  296
ident ||||||||||||||||||||||||||||||||||||||||||||||||||||||||||||
G     LCFATDVWMDEHLAKECGYTEFYYHCVQIAQRMERISPRREEYYLLKALLLANCDILLDD  297
DSSP  LLLLLLLLLLLHHHHLLLLHHHHLLLLLLHHHHHHHLLLHHHHHHHHHHHHHLLLLLLLL

DSSP  HHHHHHHHHHHHHHHHHHHHHHLLLLLLHHHHHHHLHHHHHHHHHHHHHHLLLLLLLLLL
wt    QSSLRAFRDTILNSLNDVVYLLRHSSAVSHQQQLLLLLPSLRQADDILRRFWRGIARDEV  356
ident ||||||||||||||||||||||||||||||||||||||||||||||||||||||||||||
G     QSSLRAFRDTILNSLNDVVYLLRHSSAVSHQQQLLLLLPSLRQADDILRRFWRGIARDEV  357
DSSP  HHHHHHHHHHHHHHHHHHHHHHLLLLLLHHHHHHHLHHHHHHHHHHHHHHLLLLLLLLLL

DSSP  LLLLLHHHHLL
wt    ITMKKLFLEML  367
ident |||||||||||
G     ITMKKLFLEML  368
DSSP  LLLLLHHHHLL
```
