# Supplementary material for: A Glycine Insertion in the Estrogen-Related Receptor (ERR) Is Associated with Enhanced Expression of Three Cytochrome P450 Genes in Transgenic Drosophila melanogaster
Source: PLoS One. 2015 Mar 11;10(3):e0118779. doi: 10.1371/journal.pone.0118779 (PMC4356566; doi:10.1371/journal.pone.0118779)
Supplement: S1 Table — (PDF) [file pone.0118779.s004.pdf]

Table S1. Primer Sequences for ERR cloning, sequencing, qRT-PCR and genomic amplification of LBD of *ERR*.

Gene Name	Accession number	Primer sequence (5'-3')
ERRa_fF2	NM-168258.3	ATCCAAGGCACGAACATGTCCGAC
ERR_fR2	NM-168258.3	TTGGGCGCCCGCATAATCCT
ERRb_fF2	NM-139926	CGACCACCGATGAAGTTCTACGCG
ERR_bF531	NM-139926	AAACGCACCATCCAAGGCAA
ERR_bF1032	NM-139926	TGGGCCAAGCAGATACCTGG
ERR_bR1092	NM-139926	ATCTCTGCCCACGACACCTG
ERR_bR591	NM-139926	GCCTTGCGTCTCCGCTTGTT
rp49	U92431	CGGTTACGGATCGAACAAGCG
		TTGGCGCGCTCGACAATCT
Cyp6a2	NM-078904	TTCACCACCGATGTGATTGGC
		TCGGGCATCATGCGCATT
Cyp6g1	NM-136900	AACTCCTTTGGGATGCACCTATCG
		GGTTGTGGAACCGATTGAAGTCCT
Cyp6g2	NM-136900	TCACCGCCGGCTTTGAGTCC
		GCGCAGCACCTCCAGCAGAA
Cyp9c1	NM-079126	GAGCAGCTGGGTGAGAAGCCAC
		GGCATCCACACCAGCGAATAGG
ERR-F1063	NM-168258.3	GCTTGGATCGAGTTCGTGGAG
ERR-R1613	NM-168258.3	TCTGGACGCAGTGGTAGTAGAAC
