# Supplementary material for: A Glycine Insertion in the Estrogen-Related Receptor (ERR) Is Associated with Enhanced Expression of Three Cytochrome P450 Genes in Transgenic Drosophila melanogaster
Source: PLoS One. 2015 Mar 11;10(3):e0118779. doi: 10.1371/journal.pone.0118779 (PMC4356566; doi:10.1371/journal.pone.0118779)
Supplement: S2 Table — Different programs, JASPAR [43], PROMO [44], Genome Surveyor [45], Math (TRANSFAC) (http://www.bioinfo.de/isb/gcb01/poster/index.html) and MEME [46], were used as motif discovery tools to analyze the 10 kb upstream region of a set of P450s. Conservation analyses of the putative ERR binding site regions in 12 Drosophila species were performed through UCSC Genome Browser [47]. Coordinates for the putative ERR binding sites are referred to Drosophila genome R6.01. The logos were generated using WebLogo software [48]. The logo height of the letter indicates the probability of appearing at the position in the motifs. A consensus ERR binding motif was generated using the sequences of the predicted sites for the up-regulated P450s in the 12 Drosophila species. (PDF) [file pone.0118779.s005.pdf]

P450	Aligment block window (25 bp) (strand +)/ Putative Binding Site	Site Position (Assembly R6)	Logo (Berkley edu)	Consensus motif
Cyp12d1-d	*D. melanogaster* TTTGTC--TGTTGACCTTCATGACGTC<br>*D. simulans* TTTGTC--TGTTGACCTTCATGACGTC<br>*D. sechellia* TTTGTC--TGTTGACCTTCATGACGTC<br>*D. yakuba* TTTGTC--TGTTGACCTTCATGACGTC<br>*D. erecta* TTTGTC--TGTTGACCTTCATGACGTC<br>*D. ananassae* TTTGTC--TGTTGACCTTCATGACGTC<br>*D. pseudoobscura* TTTGTT--TGTTGACCTTCATGACGTC<br>*D. persimilis* TTTGTT--TGTTGACCTTCATGACGT-<br>*D. willistoni* TCTGTA--TGTTGACCTTCATGACGTC<br>*D. virilis* TTTGTG--TGTTGACCTTCATGACGTC<br>*D. mojavensis* TGTGCGTATGTTGACCTTCATGACGTC<br>*D. grimshawi* TGTGTG--TATTGACCTTCATGACGT-	2R:11130444-11,130,451	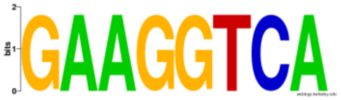	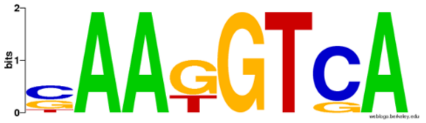
Cyp6g2	*D. melanogaster* TACCGGGCTTGACCTTGCGGTTTAG<br>*D. simulans* TGCCGGGCTTGACCTTGCGGTTTAG<br>*D. sechellia* TGCCGGGCTTGACCTTGCGGTTTAG<br>*D. yakuba* TGCCGGGCTTGACCTTGCGGTTTAG<br>*D. erecta* TGCCGGGCTTGACCTTGCGGTTTAG<br>*D. ananassae* TGCCGGGCTTGACCTTACGGTTCAA<br>*D. pseudoobscura* CGCCTGGCTTCACCTTGCGGTTTAG<br>*D. persimilis* CGCCAGGCTTCACCTTGCGGTTTAG<br>*D. willistoni* CGCCGGGTTTCACCTTACGGTTTAG<br>*D. virilis* CGCCGGGCTTGACCTTACGGTTTAG<br>*D. mojavensis* TGCCGGGCTTCACCTTACGGTTTAG<br>*D. grimshawi* TGCCTGGCTTCACCTTACGGTTTAG	2R:12200189-12,200,196	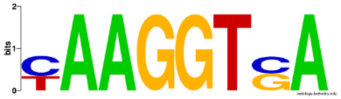	
Cyp9c1	*D. melanogaster* CATTGCGGTTGACATTGATGTAGCG<br>*D. simulans* CATTGCGGTTGACATTGATGTAGCG<br>*D. sechellia* CATTGCGGTTGACATTGATGTAGCG<br>*D. yakuba* CATTGCGGTTGACATTGATGTAGCG<br>*D. erecta* CATTGCGGTTGACATTGATGTAGCG<br>*D. ananassae* CATTGCGGTTGACATTGATGTAGCG<br>*D. pseudoobscura* CATTGTGGTTGACATTGATGTAGCG<br>*D. persimilis* CATTGTGGTTGACATTGATGTAGCG<br>*D. willistoni* CATTGCGGTTGACATTGATGTAG--<br>*D. virilis* CATTGCGGTTGACATTGATGTAGCG<br>*D. mojavensis* CATTGCGGTTGACATTGATGTAGCG<br>*D. grimshawi* CATTGCGGTTGACATTGATGTAGCG	2R:24624131-24624138	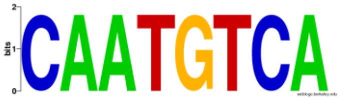	
